# Queckenstedt's Test Affects More than Jugular Venous Congestion in Rat

**DOI:** 10.1371/journal.pone.0059409

**Published:** 2013-03-13

**Authors:** Chi-Hsiang Chou, Ming-Luen Doong, Jong-Ling Fuh, Jaw-Ching Wu, Shuu-Jiun Wang

**Affiliations:** 1 Department of Neurology, Taichung Veterans General Hospital, Taichung, Taiwan; 2 Department of Neurology, Taipei Veterans General Hospital Su-Ao and Yuanshan Branch, Yi-Lan, Taiwan; 3 Department of Neurology, National Yang-Ming University School of Medicine, Taipei, Taiwan; 4 Department and Institute of Physiology, National Yang-Ming University, Taipei, Taiwan; 5 Neurological Institute, Taipei Veterans General Hospital, Taipei, Taiwan; 6 Department of Neurology, Institute of Brain Science and Brain Research Center, National Yang-Ming University School of Medicine, Taipei, Taiwan; 7 Department of Medical Research and Education, Taipei Veterans General Hospital, Taipei, Taiwan; 8 Institute of Clinical Medicine, National Yang-Ming University, Taipei, Taiwan; National Institutes of Health, United States of America

## Abstract

Jugular venous compression by the Queckenstedt's test (Q-test) increases the intracranial pressure, but the effects of isolated jugular venous congestion are not well known. Intraventricular pressure (IVP) was compared during direct obstruction of the common jugular veins (bilateral CJV clipping) and during external compression of bilateral CJV flows (Q-test) in a rat model. Intracerebroventricular catheters were inserted into the right lateral ventricle of nine male Sprague-Dawley rats (371.1±44.8 g, 82.2±12.0 days old). The initial mean IVP, arterial pressure (MAP), and pulse rate were 2.8±1.3 mmHg, 88.8±12.7 mmHg, and 348.3±69.1 beats/min, respectively. The mean IVP increment and MAP decrement were 6.5±2.5 and 13.5±5.7 mmHg, respectively, during the Q-test, compared to 2.3±1.5 and 7.3±3.8 mmHg, respectively, during bilateral CJV clipping (all *p* = 0.008). The IVP increment and MAP decrement were greater during the Q-test than during bilateral CJV clipping (*p* = 0.008 and *p* = 0.038). Although the Q-test and bilateral CJV clipping showed similar effects, the response with the Q-test was greater. Thus, the Q-test appears to obstruct other collateral cerebral veins in addition to bilateral CJV flows. Since this model revealed significant differences between the manual Q-test and bilateral CJV clipping, the finding should be taken into account in future studies on the Q-test in SD rats.

## Introduction

Queckenstedt's test (Q-test) was developed by Hans Queckenstedt in 1916 to detect spinal canal blockage by compressing the neck region just beyond the bilateral internal jugular veins [1]. This maneuver obstructs the cephalic venous return, increasing the venous blood volume in the cranial cavity and simultaneously reducing the space for cerebrospinal fluid, which in turn increases the intracranial pressure [1]–[3]. In addition to diagnosing blockage of the spinal canal, the Q-test is also used clinically to study sensitization responses in migraine patients [4]–[7].

In order to further elucidate in relationship between Q-test and sensitization responses in migraine, the establishment of a Q-test animal model should be important. Although animal studies have been performed in cats and rhesus monkeys [8], [9], the responses of rats to the Q-test and the effect of isolated bilateral jugular venous congestion are not known. Since rat is much more economic and easier to acquire than cat or rhesus monkey, to realize the intracranial pressure changes by the Q-test in rat should be of concern.

In fact the cerebral venous system of the rats is somewhat different from humans [10], because the internal jugular vein is a rather thin vessel and the external jugular vein is the main vessel for cerebral venous drainage [10]. Furthermore, the internal and external jugular veins of the rats converge to form the common jugular vein [11], [12], which does not exist in humans. Q-test is performed by external compression of bilateral common jugular veins (CJV), thus, we also wonder the effect of bilateral CJV clipping is similar or different from the Q-test in rat. The purpose of this study was to compare changes in the cerebrospinal fluid pressure by the Q-test (external compression of bilateral CJVs) and by isolated bilateral CJV clipping (direct obstruction of CJV flows) in a rat model of the Q-test. By this study, we will realize the intracranial pressure changes in the Q-test and isolated bilateral CJV clipping, and a rat Q-test model would be established.

## Materials and Methods

### Ethics Statement

Animal protocols were approved by the Institutional Animal Care and Use Committee of National Yang-Ming University. Humane care for all animals was observed, in compliance with the *Principles of Laboratory Animal Care and the Guide for the Care and Use of Laboratory Animals* (National Science Council, Taiwan, R.O.C.). The IACUC permit number was 981254.

### Animals

Adult male Sprague-Dawley (SD) rats (300–450 g) were housed individually in plastic cages in an air-conditioned room (22±1°C) under a 14-h light (6 a.m. to 8 p.m.)/10-h dark cycle. Rat chow and tap water were given ad libitum. The processes such as surgery, study procedures and sacrifice the rats were under well anesthetized conditions.

### Surgical procedures

Stainless steel cannulae were implanted in the right lateral ventricle of SD rats. Intraperitoneal sodium pentobarbital (30 mg/kg body weight, Nembutal; Abbott Laboratories, Abbott Park, IL, USA) was used for anesthetization. An anesthetized animal was placed in a stereotaxic frame (Benchmark, myNeuroLab, St. Louis, MO, USA). A stainless steel guide cannula (550 μm outer diameter [OD], 10 mm length, Elicom, Kyoto, Japan) was stereotaxically implanted according to following coordinates: 0.8 mm posterior to the bregma, 1.4 mm lateral to midline, and 2.0 mm below the surface of the right cortex, such that the cannula tip was 1.0 mm above the right cerebral ventricle [13]. Two stainless steel anchoring screws (Elicom) were fixed to the skull, and acrylic dental cement was added to secure the cannula. The SD rats were returned to their plastic cages, where they recovered for at least 7 days, with daily handling.

### Intraventricular pressure measurement

The intraventricular pressure (IVP) was recorded by the Power lab 4/30 data acquisition system (PowerLab, ADInstruments Pty Ltd., Sydney, Australia). Briefly, a stainless steel needle with a plastic cannula filled with water in a transducer (MLT0380/D Reusable BP Transducer, ADInstruments Pty Ltd) connected to the data acquisition system was inserted into the stainless needle cannula previously set over the head of the animal. The IVP of the SD rat was measured before and after the animal was anesthetized with 1.1±0.1 mg/kg urethane (Sigma, St. Louis, MO, USA). After anesthesia, rats were intubated via a catheter (PE-205, inner diameter: 1.67 mm, OD: 2.42 mm, Clay-Adam, Parsippany, NJ, USA).

### Measurement of mean arterial pressure and pulse rate

A plastic cannula filled with normal saline and heparin (10 IU/mL, Sigma) connected to another MLT0380/D transducer on a different channel of the Power lab 4/30 data acquisition system was inserted into the left or right femoral artery of the rat while in the supine position. The mean arterial pressure (MAP) and pulse rate of the femoral artery were recorded.

### Q-test

External bilateral CJV compression was performed with the index and middle fingers at the level of the bilateral submandibular glands of the rats for about 1 minute. The IVP, MAP, and pulse rates of the rats were recorded before, during, <30 sec after and >30 sec after the Q-test, which was repeated at least 3 times with each rat to confirm reproducibility. The time intervals between each Q-test (Q-test interval) were also recorded.

### Isolating and clipping of the bilateral CJVs

After the Q-test, the rat necks were dissected and the bilateral CJVs were isolated at the level of the bilateral submandibular glands. Two forceps were used to clip the bilateral CJVs at the level of the bilateral submandibular glands for about 1 minute. The IVP, MAP, and pulse rates were recorded before, during, <30 sec after clipping and >30 sec after clipping. The procedures were repeated at least 3 times in each rat to confirm reproducibility. The time intervals between the Q-test and CJV clipping (Q-test-CJV interval) and between each CJV clipping (CJV clipping interval) were also recorded.

### Crystal violet injection

To confirm the insertion position of the intracerebroventricular (ICV) cannulae, after the experiment, each animal was sacrificed by intraperitoneal injection of sodium pentobarbital (150 mg/kg). Each animal was injected with about 0.5 mL of crystal violet solution (0.05% cresyl violet, Sigma) via a stainless steel needle (Elicom). The skull was removed, and the lateral ventricle was exposed by a scalpel. If crystal violet solution was detected in the ventricle, then the ICV cannula was considered to have been positioned correctly and the animal was included in the study analyses.

### Statistical analysis

All data were calculated individually. Data are presented as means ± standard deviations (SDs). Friedman's test was used to compare the lengths of the Q-test interval, Q-test-CJV interval and CJV clipping interval. Paired sample Wilcoxon signed rank test was used to analyze changes in the IVP, MAP, and pulse rates. Statistical significance was set at *p*<0.05.

## Results

Nine male rats completed the study (371.1±44.8 g, 82.2±12.0 days old). The mean time from ICV cannula insertion to the test was 7.1±0.3 days, and the mean urethane dosage was 1.1±0.1 mg/kg. Initial and post anesthesia (before the Q-test) mean IVPs were 2.8±1.3 and 3.4±2.0 mmHg, respectively (*p* = 0.23). The initial (before the Q-test) MAP and mean pulse rates were 88.8±12.7 mmHg and 348.3±69.1 beats/min, respectively. However the mean IVP, MAP and mean pulse rates before bilateral CJV clipping and the p values in comparison with the results before the Q-test were 6.9±3.1 mmHg (*p* = 0.008), 97.2±11.2 mmHg (*p* = 0.086) and 392.3±57.1 beats/min (*p* = 0.086). The mean Q-test interval was 82.0±44.3 s, Q-test-CJV interval 683.6±386.6 s and CJV clipping interval 148.8±96.0 s. The lengths of the time intervals were different (*p* = 0.003).

**Figure 1:**
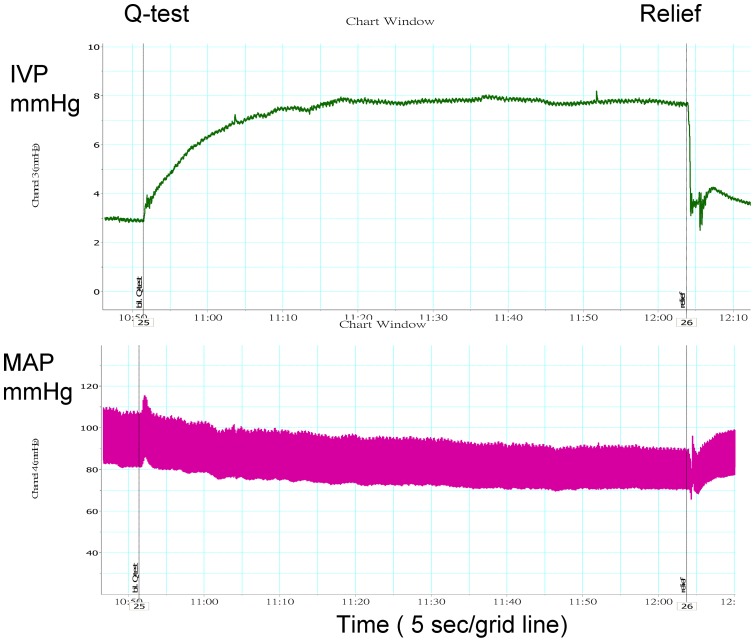
Changes in the intraventricular pressure (IVP) and mean arterial pressure (MAP) of SD rats due to Queckenstedt's test (Q-test).

**Figure 2:**
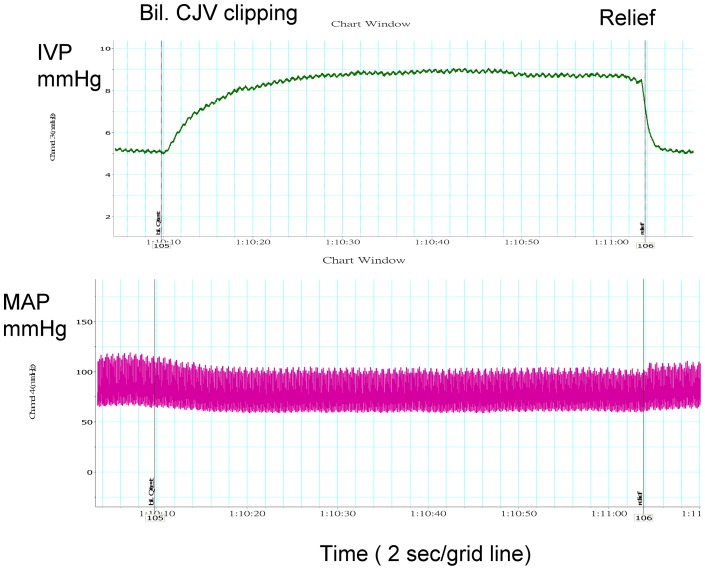
Changes in the intraventricular pressure (IVP) and mean arterial pressure (MAP) of SD rats due to bilateral common jugular venous (Bil. CJV) clipping.

The IVP increased and MAP decreased during the Q-test (Figure 1) and clipping of the bilateral CJVs (Figure 2). Table 1 shows increased IVP (9.9±2.2 vs. 3.4±2.0 mmHg, *p* = 0.008) and decreased MAP (75.3±16.7 vs. 88.8±12.7 mmHg, *p* = 0.008) during the Q-test. The IVP and MAP <30 sec after test Q-test did not differ from those before Q-test (p = 0.953, 0.314, respectively), but the pulse rate was decreased (337.0±76.1 vs. 348.3±69.1 beats/min, p = 0.028). The IVP, MAP and pulse rate >30 sec after the Q-test, however, were similar to the values before the Q-test (p = 0.594, 0.953 and 0.173, respectively).

**Table 1:** Changes in intraventricular pressure, mean arterial pressure, and pulse rate due to Queckenstedt's test.

Time point	IVP (mmHg)	*p-value*	MAP (mmHg)	*p-value*	PR (beats/min)	*p-value*
Before Q-test	3.4±2.0		88.8±12.7		348.3±69.1	
During Q-test	9.9±2.2	0.008	75.3±16.7	0.008	341.2±80.5	0.515
<30sec After Q-test	3.5±1.8	0.953	87.0±13.7	0.314	337.0±76.1	0.028
>30sec After Q-test	3.5±2.3	0.594	89.0±12.0	0.953	319.3±47.2	0.173

(n = 9).

<30 sec After Q-test: less than 30 seconds after Q-test.

>30 sec After Q-test: more than 30 seconds after Q-test.

*Abbreviations*: IVP, intraventricular pressure; MAP, mean arterial pressure; PR, pulse rate; Q-test, Queckenstedt's test. For *p*-values, comparison is to the initial time point (before Q-test).

Table 2 shows increased IVP (9.2±3.5 vs. 6.9±3.1 mmHg, *p* = 0.008) and decreased MAP (90.0±10.2 vs. 97.2±11.2 mmHg, *p* = 0.008) during bilateral CJV clipping. Pulse rate changes were insignificant in the procedure (*p* = 0.678). Both <30 sec after and >30 sec after bilateral CJV clipping, the mean IVP, MAP and pulse rate were similar to those before bilateral CJV clipping. The IVP increment and MAP decrement were greater during the Q-test than during bilateral CJV clipping (*p* = 0.008 and *p* = 0.038, respectively, Table 3).

**Table 2:** Changes of the intraventricular pressure, mean arterial pressure, and pulse rate due to bilateral common jugular venous clipping.

Time point	IVP (mmHg)	*p-value*	MAP (mmHg)	*p-value*	PR (beats/min)	*p-value*
Before	6.9±3.1		97.2±11.2		392.3±57.1	
During	9.2±3.5	0.008	90.0±10.2	0.008	395.1±48.8	0.678
<30sec After	6.8±3.0	0.110	96.3±10.2	0.441	393.0±50.7	0.859
>30sec After	7.4±3.4	0.767	97.0±8.7	0.953	377.0±42.1	0.260

(n = 9).

<30 sec After: less than 30 seconds after bilateral common jugular venous clipping.

>30 sec After: more than 30 seconds after bilateral common jugular venous clipping.

*Abbreviations*: IVP, intraventricular pressure; MAP, mean arterial pressure; PR, pulse rate. For *p*-values, comparison is to the initial time point (before bilateral common jugular venous clipping).

**Table 3:** Comparison of changes in the intraventricular pressure, mean arterial pressure, and pulse rate between Queckenstedt's test and bilateral common jugular venous clipping.

Parameter	Bilateral CJV clipping	Q-test	*p*-value
IVP changes (mmHg)	2.3±1.5	6.5±2.5	0.008
MAP changes (mmHg)	−7.3±3.8	−13.5±5.7	0.038
PR changes (beats/min)	2.8±17.9	−7.1±18.7	0.314

(n = 9).

*Abbreviations*: IVP, intraventricular pressure; MAP, mean arterial pressure; PR, pulse rate; Q-test, Queckenstedt's test; CJV, common jugular vein. For *p*-values, comparison is to the difference between the Q-test and bilateral CJV clipping.

## Discussion

We successfully demonstrated an increase in IVP in SD rats with the Q-test and bilateral CJV clipping. Our results differ somewhat from those in previous studies in cats and monkeys [8]. We observed a mean IVP of 2.8±1.3 mmHg in rats before anesthesia, compared to 3–4 mmHg in cats and 6–14 mmHg in monkeys. During the Q-test, the IVPs of SD rats and monkeys were increased. The SD rats showed decreased MAPs during the Q-test, but the change of the MAPs in monkeys was not significant. However, abdominal compression in monkeys increased IVP from 14 to 22 mmHg and decreased arterial pressure [8]. These changes during abdominal compression in the monkey study were similar to our Q-test and bilateral CJV clipping results in SD rats.

The mean IVP before bilateral CJV clipping was higher than the results before the Q-test (6.9±3.1 vs. 3.4±2.0 mmHg, *p* = 0.008), the MAP and pulse rate were increased with a trend of significance (97.2±11.2 vs. 88.8±12.7 mmHg, *p* = 0.086; 392.3±57.1 vs. 348.3±69.1 beats/min, *p* = 0.086). The causes of results above were not known. But according to Table 1 and Table 2, IVP, MAP and pulse rates returned to baseline after the Q-test for more than 30 s. The lengths of the time intervals (Q-test interval: 82.0±44.3 s; Q-test-CJV interval: 683.6±386.6 s; CJV clipping interval: 148.8±96.0 s) were different (*p* = 0.003). The reasons for the discrepancy included the more complicated procedures for surgical intervention and preparation for repeated CJV clipping during the Q-test-CJV interval and CJV clipping interval. In addition, we didn't perform any procedure in both the Q-test interval and CJV clipping interval, but surgical intervention was done in the Q-test-CJV interval. Thus we believe the longer time interval and the stress from the surgical processes to dissect and isolate the rats' bilateral CJV might explain in part the IVP differences and borderline differences of MAP and pulse rates before the Q-test and the CJV clipping. However, to confirm this point, a control group should be involved. Furthermore, the other possible cause for the baseline difference between the Q-test and bilateral CJV clipping is that during the Q-test but not CJV clipping, glomus caroticum cells might also be stimulated, resulting in the decrease of the MAP and pulse rates initially and then sympathetic reaction, so the IVP, MAP and pulse rates increased thereafter. These responses may explain in part the differences in baseline IVP, MAP and baseline of both experiments. This assumption is also partially supported by the immediate reduction in pulse rates after the Q-test when compared to that before the Q-test (337.0±76.1 vs. 348.3±69.1 beats/min; *p* = 0.028, Table 1). However, the mean pulse rates immediately after CJV clipping did not differ from those before CJV clipping (393.0±50.7 vs. 392.3±57.1 beats/min; *p* = 0.859, Table 2).

Both the Q-test and bilateral CJV clipping caused a reduction in MAP. However, in humans, although the intracranial pressure is increased by the Q-test [1], [3], MAP does not change [14]. Interestingly, previous results have shown that the MAP does not change during the Q-test in humans and monkeys, which differs from our results in rats. One possible reason for this discrepancy may be that autoregulation in the rat, as a nonerect creature, results from the autonomic nervous system and might not be as effective as that in humans. Thus, during the Q-test, although the venous returns are decreased in both species, MAP is decreased in rats but not in humans.

The pulse rate did not change significantly during the Q-test or bilateral CJV clipping. This result might be a complex finding due to the increased IVP and reduced cardiac output (caused by decreased venous return). The increased IVP may induce bradycardia [15], whereas the reduced cardiac output causes tachycardia. The effects antagonize each other, such that the net pulse rates do not change significantly.

The effects of the Q-test and bilateral CJV clipping were in the same direction in SD rats; however, the Q-test resulted in larger IVP and MAP changes than bilateral CJV clipping. We provide 2 possible explanations for this finding. First, because the trends in the IVP, MAP, and pulse rate results were similar between the Q-test and bilateral CJV clipping, the effects of the Q-test should be predominantly due to cerebral venous congestion, which increased the IVP. Second, the greater response in the Q-test suggests that, for SD rats, in addition to bilateral CJVs, there might be other collateral cerebral venous returns (as in human [16]) obstructed during the manual external compression. Therefore, more venous blood is congested in the brain, which results in higher IVP and lower MAP in the Q-test compared to direct clipping.

Our study had some limitations. First, we performed the Q-test manually. Although the test was done by the same investigator (CCH), who tried to maintain the same strength each time, variations might be unavoidable. Second, the Q-test and jugular vein clipping had to be done after urethane injection; thus, the influence of urethane could not be totally excluded. However, because both procedures were performed under similar conditions, we believe that the influence of urethane was reduced as much as possible. Third, due to technical difficulties, we cannot perform the Q-test after dissecting and isolating the rats' bilateral CJV, so we are only able to get the results of the Q-test before the surgical procedures above. The possibility of the Q-test impact on the CJV clipping cannot be totally excluded. Using two independent animal groups might solve this problem; however, the difficulties in performing the Q-test after CJV procedures should be taken into consideration. Hence, the impact of CJV procedures on the subsequent Q-test might be much greater due to more complicated procedures and technical difficulty. Before we did the surgical procedures and CJV tests, the parameters (IVP, MAP and pulse rates) had to return to the baseline after the Q-test for more than 30 sec (Table 1); therefore, we believe the Q-test impact on the CJV procedures was minimized. In addition, although the intracranial pressure increased by a significant increment after the surgical procedures, the MAP and pulse rates were only increased with borderline significance. Thus, we believe the responses to the Q-test and bilateral CJV clipping might be insignificantly influenced by the surgical process. Fourth, in this study, the regression to the mean (RTM) should be considered at the measurement of the changes of IVP, MAP and pulse rates during the intervention of the Q-test or clipping of the bilateral CJV. The best method to deal with the RTM is to design a sham test, that is, performing a similar compression as the Q-test at the vicinity of CJV when measuring the effects on IVP, MAP and pulse rates. However, since the neck of the SD rat is small compared to human fingers. It's not possible to perform such a task without involving the CJV. Nevertheless, in this study, the effects on IVP and MAP between the Q-test and bilateral CJV clipping were different. The Q-test and bilateral CJV clipping can represent the comparison groups for each other. Since a higher response of the Q-test might be through the compression of collateral vessels, it should take into account the effects of the collateral vessels when a sham test is performed. Therefore, the effect of RTM could be minimized because we compared the Q-test and bilateral CJV clipping.

Clinical applications of the Q-test have recently been increasing, especially in studies of migraine [4]–[7]. If the Q-test is performed during a migraine attack, the headache intensity may be aggravated. This so-called “Q-test headache response” has been related to increased jugular venous flow volume and could be used as a research tool to study the peripheral sensitization of migraine [7]. In such situations, a Q-test animal model might play an important role in future investigations. Our SD rat Q-test model revealed important differences between the manual Q-test and bilateral CJV clipping, which should be taken into account in future studies on the Q-test in SD rats.

## Conclusions

In rats, the effects of the Q-test versus bilateral CJV clipping were similar (increased IVP and reduced MAP), but the Q-test response was greater. In addition to bilateral CJVs, other collateral cerebral venous returns might also be obstructed during a Q-test.
